# Aldo-keto Reductase 1B10 Restrains Cell Migration, Invasion, and Adhesion of Gastric Cancer via Regulating *Integrin Subunit Alpha 5*

**DOI:** 10.5152/tjg.2023.22555

**Published:** 2023-12-01

**Authors:** Haibo Yao, Junfeng Hu, Yanfei Shao, Qinshu Shao, Shusen Zheng

**Affiliations:** 1 Department of Hepatobiliary & Pancreatic Surgery, The First Affiliated Hospital, Zhejiang University School of Medicine, Hangzhou, China; 2 Division of General Surgery, Department of Gastrointestinal and Pancreatic Surgery, Cancer Center, Zhejiang Provincial People’s Hospital (Affiliated People’s Hospital, Hangzhou Medical College), Hangzhou, Zhejiang, China; 3 Department of Pharmacy, Zhejiang Provincial People’s Hospital (Affiliated People’s Hospital, Hangzhou Medical College), Hangzhou, Zhejiang, China

**Keywords:** AKR1B10, ITGA5, gastric cancer, migration, proliferation, invasion, cell adhesion

## Abstract

**Background/Aims:**

Gastric cancer is a prevalent malignancy with unfavorable prognosis partially resulting from its high metastasis rate. Clarifying the molecular mechanism of gastric cancer occurrence and progression for improvement of therapeutic efficacy and prognosis is needed. The study tended to delineate the role and regulatory mechanism of *aldo-keto reductase 1B10* (*AKR1B10*) in gastric cancer progression.

**Materials and Methods:**

The relationship of *AKR1B10* expression with survival rate in gastric cancer was analyzed through Kaplan–Meier analysis. The mRNA levels of *AKR1B10* and *integrin subunit alpha 5 (ITGA5)* in gastric cancer tissues and cell lines were measured by real-time quantitative polymerase chain reaction. Protein levels of *AKR1B10* and integrin subunit alpha 5 were assayed via western blot. The molecular relationship between *AKR1B10* and *ITGA5* was analyzed by co-immunoprecipitation assay. Cell viability was assayed through Cell Counting Kit-8, invasion and migration of tumor cells was assessed through wound healing and transwell assays. Transwell assay was utilized to detect invasion. The adhesion of gastric cancer cells was detected using cell adhesion assays.

**Results:**

The results unveiled that *integrin subunit alpha 5* was upregulated, while *AKR1B10* was downregulated in gastric cancer tissues and cells. Overexpressing *AKR1B10* hindered gastric cancer cell proliferation, migration, invasion and adhesion. It was striking that we certified the inhibitory effect of *AKR1B10* on *integrin subunit alpha 5* expression and their (*AKR1B10* and *ITGA5*)) negative relationship via bioinformatics method, real-time quantitative polymerase chain reaction, and co**-**immunoprecipitation assays. Via rescue experiments, it was concluded that *AKR1B10* served as tumor suppressor potentially by *ITGA5* expression in gastric cancer.

**Conclusion:**

Our results indicated that *AKR1B10* inhibited migration, invasion, and adhesion of gastric cancer cells via modulation of *ITGA5*.

**Main Points**

- *Aldo-keto reductase 1B10* (*AKR1B10)* was lowly expressed in gastric cancer (GC). *AKR1B10* inhibits GC cell proliferation, migration, invasion and adhesion.
- *Integrin subunit alpha 5* (*ITGA5)* is a downstream regulatory gene of *AKR1B10*. *AKR1B10* regulates *ITGA5*to function in GC cells.

## Introduction

Gastric cancer (GC) is a major cause of cancer-related deaths.^1^ Metastasis of GC at early stage likely results in poor prognosis and high mortality.^2^ The research revealed that 5-year survival rate of advanced GC patients is less than 15%.^3^ The complexity and multifactorial nature of metastasis mechanism in GC are considered as the main obstacles to promoting the survival of patients.^4^ Moreover, the molecular mechanism of GC development has not been clearly explored at present. Hence, exploring new molecular mechanisms of GC progression and finding new potential therapeutic targets are needed for improving the survival rate of GC patients. 

Aldo-keto reductase 1B10 (AKR1B10) belongs to AKR superfamily. Aldo-keto reductase family are mainly soluble monomers with a molecular weight ranging from 34 to 37 kDa, and the AKR superfamily consists of nearly 200 members that can be classified into 16 categories with different subtypes in each category.^5^ A study verified that AKR members may participate in biological processes including carbonyl detoxification, hormone and lipid metabolism, osmoregulation, tumor formation, and tumor drug resistance in the organism.^6^ Aldo-keto reductase 1B10 participates in the occurrence of various tumors. Aldo-keto reductase 1B10 level is high in non-gastrointestinal solid tumors like liver cancer,^7^ lung cancer,^8^ breast cancer,^9^ and oral squamous cell carcinoma,^10^ while it is decreased in colorectal cancer,^11^ which may be caused by the tissue specificity of AKR1B10. Aldo-keto reductase 1B10 is mainly found in distal gastrointestinal tract, such as the small intestine and colorectum of healthy people.^12^ Aldo-keto reductase 1B10 can effectively catalyze the reduction of cytotoxic unsaturated carbonyl groups, thereby protecting the gastrointestinal tract from carboxyl damage and reducing the occurrence of tumors.^13^ Hence, we can infer that AKR1B10 acts as a tumor inhibitor in the occurrence and progression of gastrointestinal tumors, which is congruous with the previous finding that AKR1B10 is low in GC.^14^ Nevertheless, the regulatory gene downstream of *AKR1B10* and its mechanism in GC cells need further exploration.

*Integrin subunit alpha 5* (*ITGA5*), a member of integrin family, is cancer-promoting by inducing communication between different cells or between cells and extracellular matrix.^15^ *ITGA5* has been shown to produce an integral membrane protein to promote breast cancer cell metastasis.^16^ *ITGA5* stimulates tumor progression and serves as an independent prognostic factor in esophageal squamous cell carcinoma.^17^ In addition, fibroblasts repressing colorectal adenocarcinoma require *ITGA5*, which is possible to be a stromal prognostic marker.^18^ But regulatory relationship of *ITGA5 *with *AKR1B10* has not been investigated. 

The main purpose of this work was to delineate expression levels of *AKR1B10*/*ITGA5* and the specific mechanism of *AKR1B10*/*ITGA5* in GC cell proliferation, migration, invasion, and cell adhesion through in vitro molecular and cellular assays, thereby assisting in finding new molecular targets for GC diagnosis and treatment.

## Materials and Methods

### Bioinformatics Method

Clinical sample profiles of The Cancer Genome Atlas–Stomach adenocarcinoma (TCGA–STAD) including 32 normal samples and 375 GC tissue samples were accessed from TCGA (https://portal.gdc.cancer.gov/) and were subjected to *t*-test for expression analysis of *AKR1B10*. The data were filtered out and processed via differential analysis by R package “edgeR” (|log fold change (FC)|>2, false discovery rate (FDR) < 0.05). Correlation analysis of AKR1B10 expression with clinical characteristics, and K-M analysis of *AKR1B10* expression level and overall survival in GC were based on the TCGA database. Survival analyses were carried out using the “survival” package.

### Clinical Samples

Gastric cancer (n = 20) and adjacent normal (n = 20) tissue samples of patients who did not undergo radiotherapy or chemotherapy before surgery were retrieved from Zhejiang Provincial People’s Hospital, with all samples stored in liquid nitrogen. The study acquired approval from the Ethics Committee of Zhejiang Provincial People’s Hospital (No: 2018KT050) and informed consent was obtained from each subject.

### Cell Culture and Transfection

Human normal gastric mucosal cell line GES-1 (bio-81778) and GC cell lines SGC-7901 (bio-73147), BGC-823 (bio-72975), MKN-45 (bio-73233), and AGS (bio-69318) were accessed from Biobw Biotechnology (Beijing, China). All cell lines were maintained in RPMI-1640 (Gibco, USA, CA, Carlsbad) plus 10% heat-inactivated fetal bovine serum (FBS) (Gibco) in a humidified incubator with 5% CO_2_ at 37°C.

SGC-7901 and AGS cells were seeded in 6-well plates (1×10^5^ cells/well) and cultured at 37°C overnight. oe-*AKR1B10*, oe-*ITGA5*, si-*AKR1B10*, and their corresponding controls were bought from GenePharma (China, Shanghai), and pcDNA3.1 vectors (Thermo Fisher Scientific, USA, MA, Waltham) were utilized as overexpression vectors. Lipofectamine 2000 (Thermo Fisher Scientific) was utilized to transfect plasmids. The 6-well plates were shaken gently to disperse complexes evenly, and cells were incubated for 24-48 hours at 37°C. Afterward, transfection efficiency was assayed via real-time quantitative polymerase chain reaction (qRT-PCR).

### Co-Immunoprecipitation

Co-immunoprecipitation was applied to measure the interaction of *AKR1B10* and *ITGA5*. SGC-7901 cells were lysed using radio immunoprecipitation assay (RIPA) buffer (Thermo Fisher Scientific) to harvest cell lysates. Next, cell lysates were maintained at 4°C overnight with anti-*AKR1B10* (ab192865, Abcam, UK), anti-*ITGA5* (ab150361, Abcam, Cambridge), or control IgG (ab172730, Abcam), followed by 1 hour of incubation with protein A/G agarose beads (Thermo Fisher Scientific) at 4°C. After beads were rinsed 4 times with pre-ice-cold lysis buffer, Sodium Dodecyl Sulfate PolyAcrylamide Gel Electrophoresis (SDS-PAGE) sample buffer was supplemented for denaturation. *AKR1B10* and *ITGA5* protein levels were assayed via western blot.

### RNA Isolation and Real-Time Quantitative Polymerase Chain Reaction

Total RNA was isolated from tissues and cell lines with Trizol reagent (Invitrogen, USA, MA, Waltham). cDNA was synthesized by PrimeScript reverse transcriptase reagent kit (TaKaRa, Japan, Kyoto). Real-time quantitative polymerase chain reaction was done on synergy brandssynergy brands (SYBR) green PCR mix (TaKaRa) with glyceraldehyde-3-phosphate dehydrogenase (GAPDH) as the internal reference. Primer sequences were listed in Table 1. Data were analyzed by applying 2^−ΔΔCt^ method.

### Western Blot

After 24 hours of transfection, cells were rinsed with phosphate buffer solution (PBS) and lysed in the lysis buffer (Cell Signaling Technology, USA, MA, Boston) for 30 minutes. Protein concentration was identified with bicinchoninic acid protein assay kit (Pierce Biotechnology, USA, IL, Rockford). Proteins were subjected to SDS-PAGE and transferred onto a membrane. The membrane was sealed with 5% skim milk and then blotted overnight with primary antibodies anti-AKR1B10 (ab192865, 1:5000, Abcam), anti-ITGA5 (ab150361, 1:5000, Abcam), and anti-GAPDH (ab181602, 1:10 000, Abcam). After rinsing, the membrane was incubated with horseradish peroxidase-coupled secondary antibody IgG H&L (ab6721, 1:2500, Abcam) at room temperature for 1 hour. Signals were developed using enhanced chemiluminescence (ECL) detection reagent (Pierce) and the gray value was assessed on Image J software (Bethesda, Md, USA). GAPDH was utilized as a protein load control. 

### Cell Counting Kit-8 Assay

The transfected SGC-7901 cells (1 × 10^3^ cells/well) were inoculated in 96-well plates for 0, 24, 48, 72, and 96 hours of incubation. Cell viability was detected with Cell Counting Kit-8 (CCK-8) kit (Dojindo, Japan, Kyushu). The optical density (OD) value at 450 nm was assayed with Elx800 Reader (Bio-Tek Instruments Inc., USA, VT, Winooski).

### Transwell Assay

For detection of cell migration, 5 × 10^4^ cells in 200 μL medium (0.1% FBS) were placed in the upper chamber of the insert (pore size 8 μm) (Becton-Dickinson Biosciences, USA, NJ, Sussex), while the lower chamber was supplemented with 600 μL medium plus 10% FBS. For detection of cell invasion, cell suspension of the same density was added in the upper chamber pre-coated with Matrigel and 600 μL medium plus 10% FBS in the lower chamber (Becton-Dickinson Biosciences) for 48 hours of incubation. After incubation, cells in the upper chamber were wiped off with a cotton swab, while cells on the other side were fixed with 4% paraformaldehyde and stained with 0.1% crystal violet in 20% ethanol. The migrating and invading cells were monitored by photographs of 5 independent fields of each well using a LEICA microscope (Germany). This determination was conducted 3 times.

### Wound Healing Assay

After transfection, cells were seeded on 6-well plates (2×10^5^ cells/well) and grew to confluence. Monolayer was scratched with a 200 μL pipette tip and the floating cells were washed off with fresh medium, the remaining cells were followed by 24 hours of cell culture in an incubator under routine conditions. The wound areas were photographed at 0 and 24 hours for calculating the cell migration rate. The wound healing rate was computed as wound healing rate (%) = (wound width at 0 hour − wound width at 24 hours)/wound width at 0 hour × 100%.

### Cell Adhesion Assay

To prevent non-specific cell adhesion, the 96-well plate was pre-treated with 10 μg/mL fibronectin (100 μL/well) (Becton-Dickinson Biosciences) at 37°C for 1 hour and treated with 1% bovine serum albumin (BSA) for 45 minutes. Firstly, cells were resuspended in a medium plus 1% FBS to adjust cell concentration. Cells were plated in the 96-well plate (2×10^4^ cells/well) for 1 hour of incubation in the optimal incubator. Then, supernatant was discarded. Finally, cells were fixed for 15 minutes with 4% paraformaldehyde, stained with 0.2% crystal violet, counted, and pictured under a microscope.

### Statistical Analysis

The relationship between *AKR1B10* and *ITGA5* in TCGA database and clinical samples was analyzed by the Pearson correlation. Measurement data were presented as the mean ± SD, and each experiment was completed at least 3 times. All data were subject to Student’s *t*-test or 1-way analysis of variance on GraphPad Prism 5.0 (GraphPad Software, Inc., USA, CA, San Diego) and were then subject to Tukey’s posthoc test for multiple comparisons. *P *< .05 means a statistically significant difference.

## Results

### *Aldo Keto Reductase 1B10* Expression Is Noticeably Lower in Gastric Cancer

**Figure 1.**
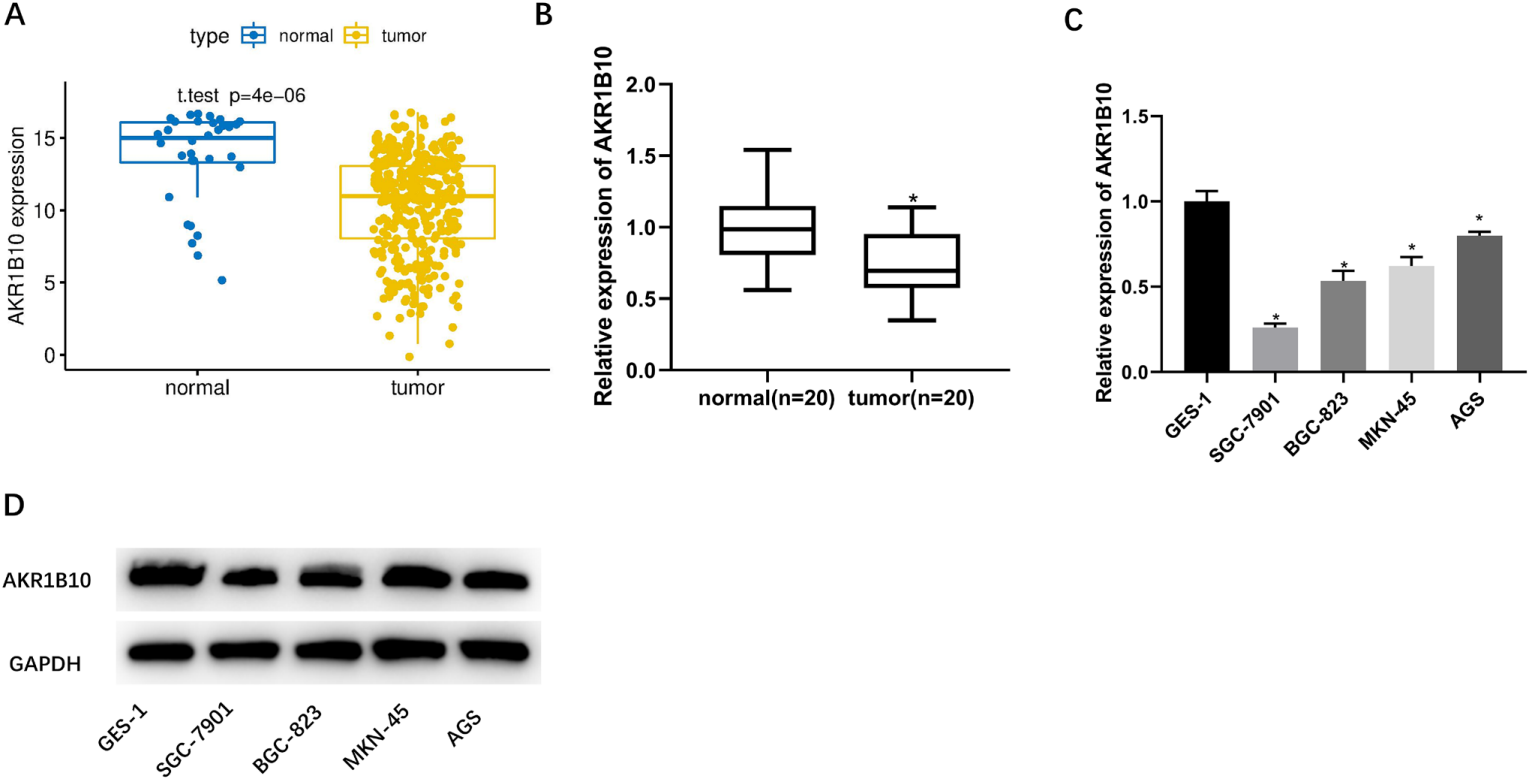
Aldo-keto reductase 1B10 is markedly lowly expressed in GC. (A) The mRNA expression level of *AKR1B10* in GC tissues and adjacent normal tissues was analyzed by TCGA database (blue: adjacent normal tissues; yellow: tumor tissues). (B) The mRNA expression level of *AKR1B10* in clinical tumor and adjacent normal tissue samples was assessed via qRT-PCR (n = 20). (C) *AKR1B10* mRNA level in GES-1, SGC-7901, BGC-823, MKN-45, and AGS cell lines assessed via qRT-PCR; (D) *AKR1B10* protein level in GES-1, SGC-7901, BGC-823, MKN-45, and AGS cell lines measured by western blot; ^*^ *P *< .05. *AKR1B10, aldo-keto reductase 1B10*; qRT-PCR, real-time quantitative polymerase chain reaction; TCGA, The Cancer Genome Atlas.

**Supplementary Figure 1.**
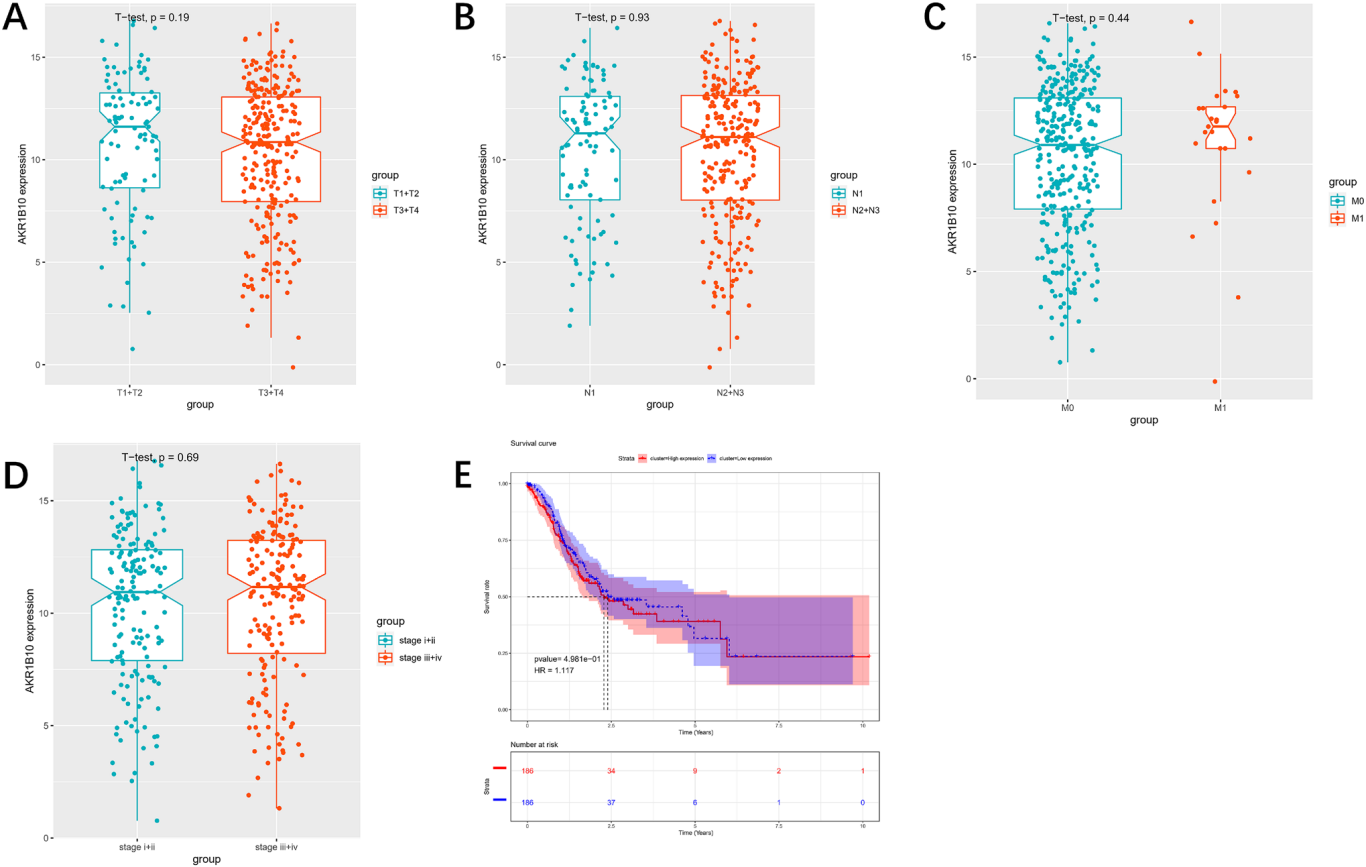
(A-D) The correlation analysis between AKR1B10 expression level and clinical characteristics in GC; (E) K-M analysis between AKR1B10 expression level and overall survival in GC.

In the previous reviews, our researchers found that *AKR1B10* is a favorable prognostic indicator in GC and remarkably downregulated in clinical samples.^14^ Therefore, this study mainly probed the specific relevant molecular mechanism of *AKR1B10* in the occurrence and metastasis of GC. Firstly, bioinformatics methods were used to analyze data in TCGA-STAD dataset. The *AKR1B10* level in GC tissues was dramatically lower than that in normal tissues (Figure 1A). Also, correlations between *AKR1B10* expression levels and clinical characteristics as well as overall survival were analyzed. The results showed no significant differences in *AKR1B10* expression among T classification (*P *= .19), N classification (*P *= .93), M classification (*P *= .44), clinical stage (*P *= .69), and overall survival (Supplementary Figure 1A-E). Real-time quantitative polymerase chain reaction was implemented to quantify *AKR1B10* level in 20 pairs of clinical samples of GC tissue and adjacent normal tissue, which manifested that *AKR1B10* level in the clinical samples of GC tissue showed the same trend as predicted by bioinformatics analysis (Figure 1B). Subsequently, mRNA and protein levels of *AKR1B10* in GES-1, SGC-7901, BGC-823, MKN-45, and AGS cell lines were assayed via qRT-PCR and western blot. The *AKR1B10* mRNA and protein levels were markedly reduced in GC cell lines (Figure 1C and  1D). Overall, *AKR1B10* in GC tissues and cells was relatively less expressed. Then, SGC-7901 cell line with the lowest level of *AKR1B10* and AGS cell line with the highest level of *AKR1B10* were chosen for subsequent experiments.

### Aldo-Keto Reductase 1B10 Restrains Cell Viability, Migration, Invasion, and Adhesion of Gastric Cancer Cells In Vitro

**Figure 2.**
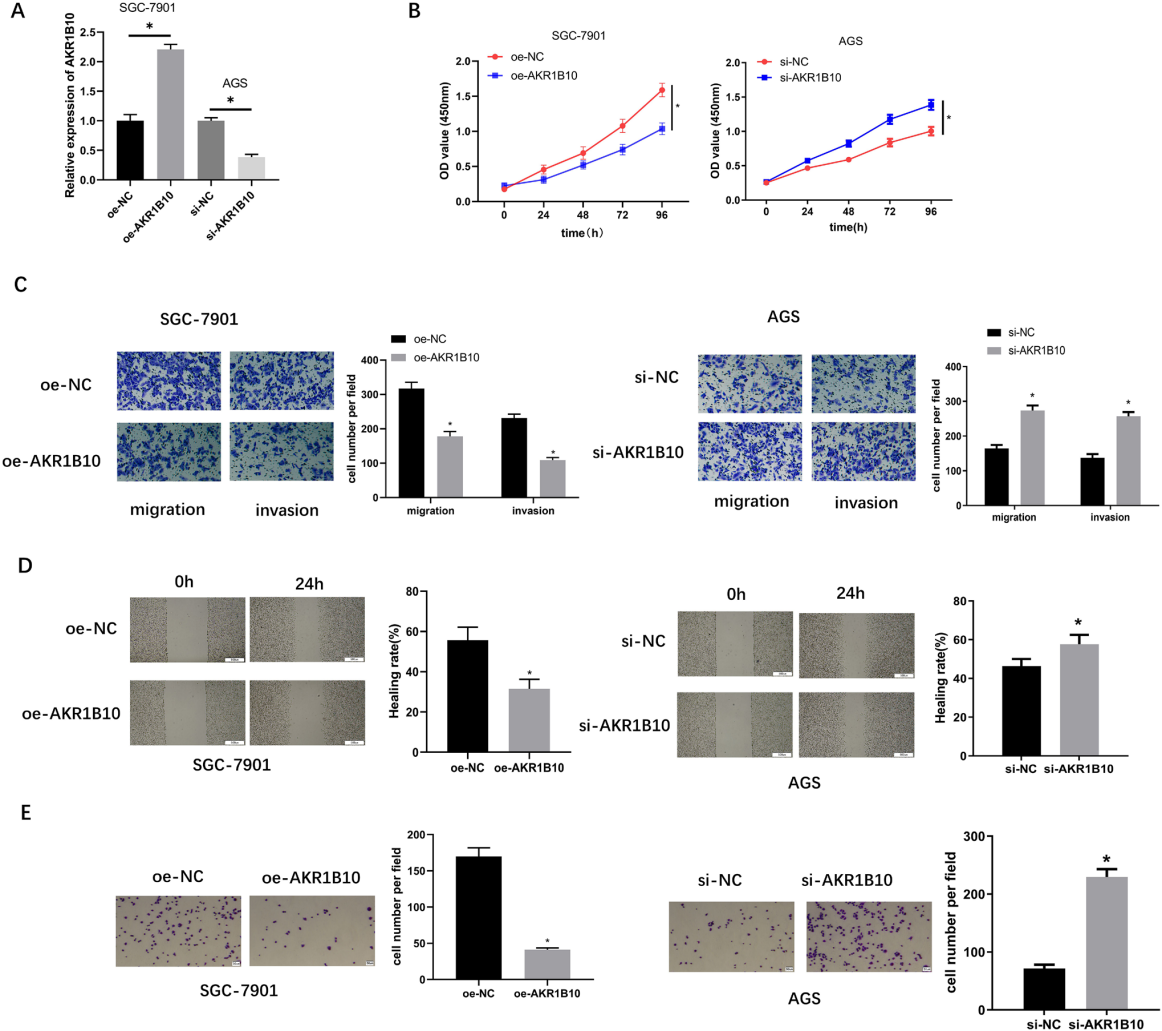
*AKR1B10* restrains cell viability, invasion, migration, and adhesion of GC in vitro. (A) *AKR1B10* mRNA expression in each transfection group was assayed via qRT-PCR; (B) GC cell viability in different treatment groups was measured via CCK-8 assay; (C) migratory and invasive abilities of GC cells in different treatment groups were assessed through transwell assays (×100); (D) migratory ability of GC cells in different treatment groups were assayed through wound healing assay (×40); (E) the adhesive ability of GC cells in different treatment groups was detected by cell adhesion assay (×100); ^*^ *P *< .05. *AKR1B10, aldo-keto reductase 1B10*; CCK-8, Cell Counting Kit-8; GC, gastric cancer; qRT-PCR, real-time quantitative polymerase chain reaction.

To measure the impact of *AKR1B10* on cell viability, migration, invasion, and adhesion of GC cells in vitro, oe-*AKR1B10* was transfected into SGC-7901 cells and si-*AKR1B10* was transfected into AGS cells, respectively. Transfection efficiencies were assayed through qRT-PCR, and results indicated that *AKR1B10* level was noticeably up-regulated after transfecting with oe-*AKR1B10* and downregulated after transfecting with si-*AKR1B10* (Figure 2A). Cell Counting Kit-8 assay verified that overexpressing *AKR1B10* remarkably inhibited cell viability, but the si-*AKR1B10* group exerted the opposite effect (Figure 2B). Transwell migration and invasion assays substantiated that overexpressing *AKR1B10* conspicuously hindered cell migratory and invasive abilities, and silencing *AKR1B10* promoted the above phenotypes (Figure 2C). As such, the wound healing assay also ascertained that overexpressing *AKR1B10* could noticeably hamper cell migratory ability (Figure 2D). Cell adhesion assay displayed that overexpressing *AKR1B10* notably reduced cell adhesive ability (Figure 2E). However, knockdown of *AKR1B10* fostered cell migration and adhesion of GC cells. Collectively, *AKR1B10* served as tumor suppressor in GC.

### Overexpression of *Aldo-keto Reductase 1B10* Inhibits the Expression of *Integrin Subunit Alpha 5*

**Figure 3.**
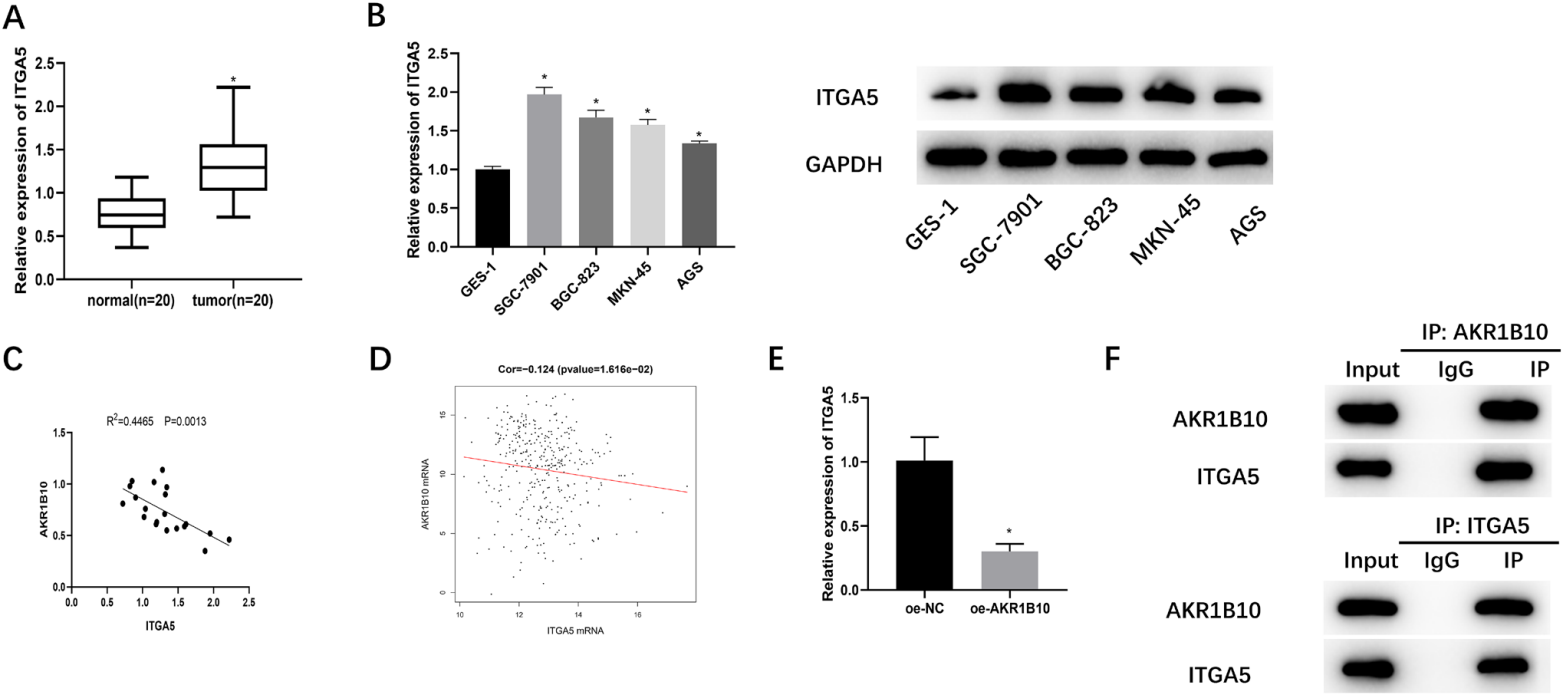
*ITGA5* expression is up-regulated in GC tissues and cells, and overexpressing *AKR1B10 *suppresses *ITGA5* expression. (A) *Integrin subunit alpha 5* mRNA level in clinical samples of GC tissues (n = 20) and adjacent normal tissues (n = 20) detected through qRT-PCR; (B) qRT-PCR and western blot detected *ITGA5* mRNA and protein levels in GES-1, SGC-7901, BGC-823, MKN-45, and AGS cell lines; (C) Correlation analysis of *AKR1B10* and *ITGA5* levels in clinical samples; (D) Correlation analysis of *AKR1B10* and *ITGA5* levels using TCGA database; (E) qRT-PCR measured *ITGA5* mRNA level of cells in each transfection group (oe-NC, oe-*AKR1B10*); (F) the protein interactions between *AKR1B10* and *ITGA5* were analyzed by Co-IP assay; ^*^ *P *< .05. *AKR1B10, aldo-keto reductase 1B10*; CCK-8, Cell Counting Kit-8; Co-IP, co-immunoprecipitation; *ITGA5, integrin subunit alpha 5*; qRT-PCR, real-time quantitative polymerase chain reaction; TCGA, The Cancer Genome Atlas.

Based on the previous research,^19^ we conjectured that *ITGA5* may be a downstream regulatory gene of *AKR1B10*. The expression analysis results of *ITGA5* in clinical samples detected by qRT-PCR were shown in Figure 3A, which denoted that *ITGA5* expression in tumor tissues (n = 20) was prominently higher than that in adjacent normal tissues (n = 20). mRNA and protein levels of *ITGA5* in GES-1, SGC-7901, BGC-823, MKN-45, and AGS cell lines were assayed through qRT-PCR and western blot, respectively. In accordance with results in clinical samples, mRNA and protein levels of *ITGA5* in GC cells were dramatically higher than in normal gastric mucosal cells (Figure 3B). In the analysis of clinical samples and TCGA database, *ITGA5* was negatively correlated with *AKR1B10* (Figure 3C and  3D). Likewise,* ITGA5* mRNA level was noticeably reduced in oe-*AKR1B10* group (Figure 3E), which suggested that *AKR1B10* suppressed the expression of *ITGA5*. Interaction of *AKR1B10* with *ITGA5* was confirmed using Co-IP assays (Figure 3F), which confirmed the binding relationship of *AKR1B10* with *ITGA5*. Thus, *ITGA5* was upregulated in GC and negatively regulated by *AKR1B10*.

### Aldo-keto Reductase 1B10 Regulates *Integrin Subunit Alpha 5* to Function in Gastric Cells

**Figure 4.**
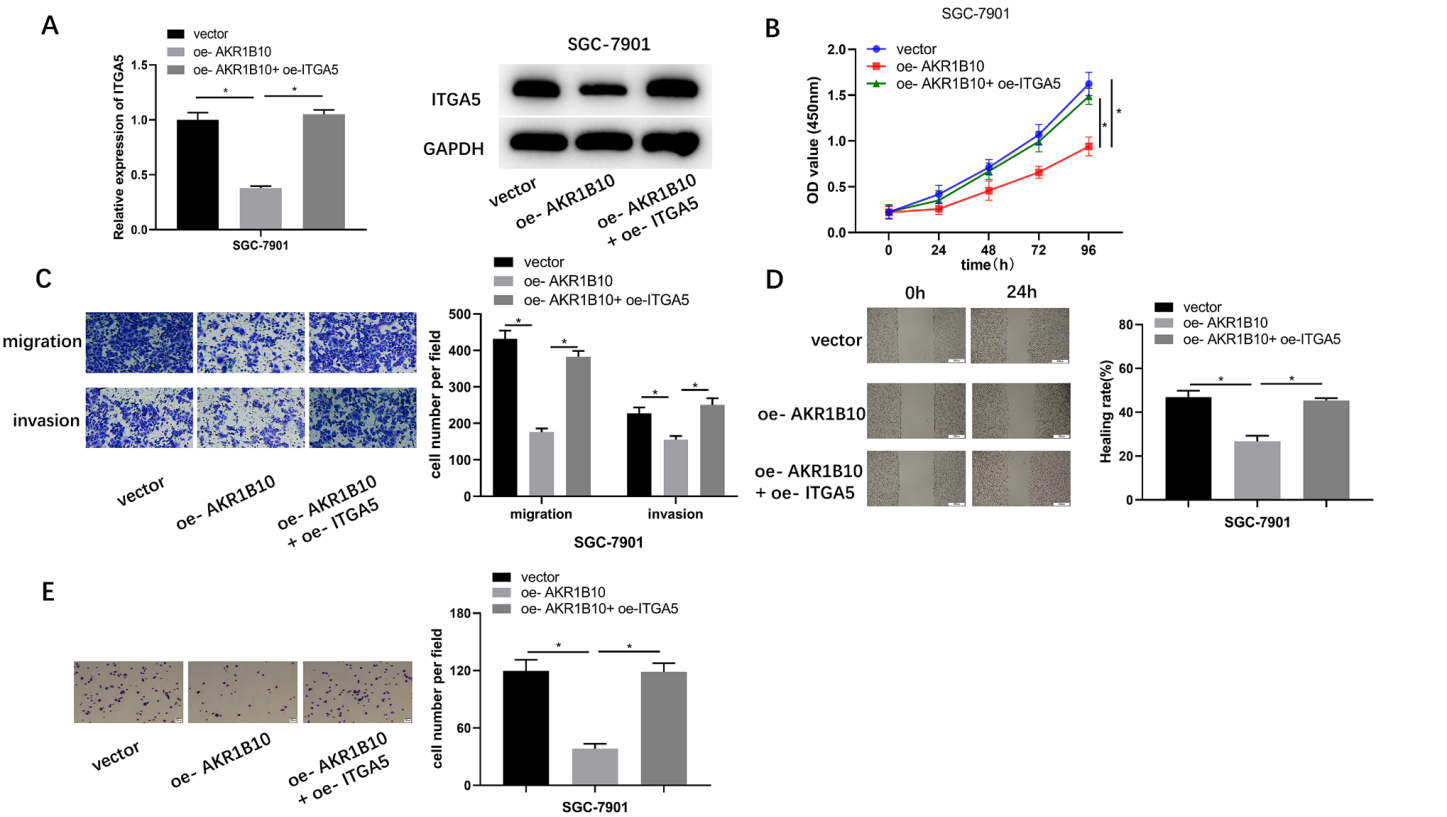
Aldo-keto reductase 1B10 regulates *ITGA5* to function in GC cells. (A) Integrin subunit alpha 5 mRNA and protein levels in different treatment groups were detected via qRT-PCR and western blot, respectively; (B) Cell viability in different treatment groups was detected via CCK-8 assay; (C) Migratory and invasion abilities in different treatment groups were tested through Transwell assay (×100); (D) Migratory ability in different treatment groups was detected via wound healing assay (×40); (E) Cell adhesive property in different treatment groups was tested by cell adhesion assay (×100); ^*^ *P *< .05. *AKR1B10, aldo-keto reductase 1B10*; CCK-8, Cell Counting Kit-8; *ITGA5, integrin subunit alpha 5*; qRT-PCR, real-time quantitative polymerase chain reaction; oe, overexpressing.

To investigate the impact of *AKR1B10* on GC cells (SGC-7901) by regulating *ITGA5*, a rescue experiment was carried out and 3 groups were constructed: vector [(overexpressing)oe-NC(*AKR1B10*)+oe-NC(*ITGA5*)], oe-*AKR1B10* [oe-*AKR1B10* + oe-NC(*ITGA5*)], and oe-*AKR1B10* + oe-*ITGA5*. Firstly, transfection efficiency was validated by qRT-PCR and western blot. Then, qRT-PCR and western blot respectively manifested that *ITGA5* mRNA and protein levels decreased substantially in oe-*AKR1B10* group, whereas their expression levels were restored in oe-*AKR1B10*+oe-*ITGA5* group (Figure 4A). Through CCK-8 assay, it was found that overexpressing *ITGA5* and *AKR1B10* simultaneously could neutralize the repressive effect of overexpressing *AKR1B10* on cell viability (Figure 4B). Migratory and invasive potentials of SGC-7901 cells were attenuated in oe-*AKR1B10* group, but this effect was offset by overexpressing *ITGA5* and *AKR1B10* simultaneously (Figure 4C and  4D). Cell adhesion assay also revealed a consistent trend that overexpressing *AKR1B10* and *ITGA5* simultaneously abrogated the inhibition of cell adhesion by overexpressing *AKR1B10* (Figure 4E). Therefore, *AKR1B10* could suppress proliferation, migration, invasion, and adhesion of GC cells by modulating *ITGA5*.

## Discussion

Since GC is a common malignancy, it is of great significance for improvement of treatment and patient’s prognosis to clarify its occurrence and progression mechanism. *AKR1B10* is known to be a good clinical and prognostic predictor for GC^14, 20^ and affects doxorubicin resistance and cisplatin resistance of GC cells.^21, 22^ Shen et al^23^ established a mice model and found increased gene mutations and dysplasia of intestinal mucosal cells in mice deficient in *AKR1B8* (the ortholog of human *AKR1B10*). Numerous studies substantiated that *AKR1B10* expression is low in GC.^24-26^ Shao et al^17^ reported that *AKR1B10* was lowly expressed in GC tissues and repressed proliferation, migration, and invasion of GC cells. This study also manifested that *AKR1B10* levels in GC tissues and cells were conspicuously lower in comparison to normal tissues and cells through analyzing TCGA-STAD dataset and detecting the *AKR1B10* expression in GC tissues and cell lines in vitro, which was in line with the earlier studies. *AKR1B10* plays a pro-cancer role in varying cancer species. Qu et al^18^ reported that *AKR1B10* was substantially upregulated in breast cancer tissues, and it facilitated cell malignant phenotypes via modulation of Phosphoinositide 3-kinase/Protein Kinase B/Nuclear factor kappa-B (PI3K/AKT/NF-κB) signaling pathway. Huang et al^27^ revealed that *AKR1B10* was highly expressed in bladder cancer tissues and modulated the proliferation, migration, invasion, and cell stemness of bladder cancer cells through the CUX7/AKR1B10/ERK signaling pathway. Liu et al^28^ demonstrated that *AKR1B10* expression was substantially associated with T-stage and clinical stage of colon cancer, and knockdown of *AKR1B10* substantially attenuated tumor cell proliferation and clonogenic capacity. In this work, we overexpressed *AKR1B10* in SGC-7901 cells and silenced *AKR1B10* in AGS cells. Through CCK-8, wound healing, transwell, and cell adhesion assays, it was confirmed that overexpressing *AKR1B10* could dramatically inhibit cell viability, migration, invasion, and adhesion of GC cells, which further supported results in previous research. 

An existing study showed that *AKR1B10* can regulate the expression of *ITGA5* in breast cancer.^19^ *ITGA5* mainly distributes in fibroblasts and is connected to fibronectin. *ITGA5* is involved in varying cancers. For instance, in esophageal squamous cell carcinoma, *ITGA5* can promote cell migration and cisplatin resistance.^29^ *ITGA5* can increase the survival rate and metastatic potential of lung cancer cells.^30^ *ITGA5* promotes GC cell proliferation, migration, invasion, and tumor growth in vivo by focal adhesion kinase (FAK)-AKT signaling pathway.^31^ *ITGA5* fosters GC cell proliferation, migration, invasion, and metastasis in vivo by inducing the epithelial-to-mesenchymal transition process.^32^ This study carried out a bioinformatics analysis to reveal that *ITGA5* was negatively correlated with *AKR1B10*, and* ITGA5* was increased in GC tissues and cells. The result was in line with a result in an earlier study.^31^ Afterward, in vitro rescue experiments in cells justified that *AKR1B10* suppressed GC cell viability, migration, invasion, and adhesion by regulating *ITGA5*, which further verified the regulatory relationship between *AKR1B10* and *ITGA5*.

However, these results had certain limitations. For instance, the regulatory mechanism between *AKR1B10* and *ITGA5* was not certified in vivo nor was the specific downstream regulatory mechanism of *ITGA5* in regulating cell biological functions. These deficiencies would be developed in further research.

## Conclusion

All in all, this study verified that *AKR1B10* was notably downregulated, while *ITGA5* was markedly upregulated in GC, and *AKR1B10* hampered GC cell viability, invasion, migration, and adhesion by regulating* ITGA5*, through analysis of TCGA data along with clinical data, as well as in vitro molecular and cell experiments. These findings lay a theoretical foundation for exploring novel molecules for clinical diagnosis and treatment of GC and generate fresh insight into finding safer and more effective treatments for GC. 

## Availability of Data and Materials:

The data used to support the findings of this study are included within the article. The data and materials in the current study are available from the corresponding author upon reasonable request.

## Figures and Tables

**Table 1.** Primer Sequences in Real-Time Quantitative Polymerase Chain Reaction

qRT-PCR Primer	Sequence (5ʹ-3ʹ)
*AKR1B10*	F: CCCAGGTTCTGATCCGTTTC
	R: GGTTGCCATCTCCTCATCAC
*ITGA5*	F: TGCAGTGTGAGGCTGTGTACA
	R: GTGGCCACCTGACGCTCT
*GAPDH*	F: CACCCACTCCTCCACCTTTG
	R: CCACCACCCTGTTGCTGTAG

*AKR1B10*, aldo-keto reductase 1B10; *ITGA5*, integrin subunit alpha 5.
